# CRISPR/Cas genome editing system and its application in potato

**DOI:** 10.3389/fgene.2023.1017388

**Published:** 2023-02-13

**Authors:** Xin Hou, Xiaomeng Guo, Yan Zhang, Qiang Zhang

**Affiliations:** College of Plant Protection, Shandong Agricultural University, Tai’an, China

**Keywords:** potato, gene editing, CRISPR/Cas, genetic improvement, progress

## Abstract

Potato is the largest non-cereal food crop worldwide and a vital substitute for cereal crops, considering its high yield and great nutritive value. It plays an important role in food security. The CRISPR/Cas (clustered regularly interspaced short palindromic repeats/CRISPR-associated) system has the advantages of easy operation, high efficiency, and low cost, which shows a potential in potato breeding. In this paper, the action mechanism and derivative types of the CRISPR/Cas system and the application of the CRISPR/Cas system in improving the quality and resistance of potatoes, as well as overcoming the self-incompatibility of potatoes, are reviewed in detail. At the same time, the application of the CRISPR/Cas system in the future development of the potato industry was analyzed and prospected.

## 1 Introduction

CRISPR/Cas is a part of the CRISPR adaptive immune system, including CRISPR and CRISPR-related protein genes (Sorek et al., 2008), that has been developed as an important tool in genome editing (Maximiano et al., 2021). Compared with transcription activator-like effector nuclease (TALEN) (Christian et al., 2010) and zinc-finger nuclease (ZFN) (Urnov et al., 2010), CRISPR/Cas has obvious advantages of simplicity, flexibility, efficiency, and economy (Cho et al., 2013; Wang et al., 2013; Castro et al., 2021). TALEN and ZFN technologies are technically difficult, making the construction of vectors time consuming; they cannot be efficiently utilized in routine laboratories, while CRISPR/Cas technologies are relatively simple to operate, making their use in the production of vectors inexpensive. They can be more widely used in general molecular biology laboratories. Therefore, CRISPR/Cas has become the most powerful genetic tool for crop character improvement and quality optimization. At present, it has been successfully applied to *Arabidopsis thaliana*, *Sorghum bicolor*, *Nicotiana tabacum*, *Oryza sativa*, and other plants (Jiang et al., 2013; Hussain et al., 2018; Jaganathan et al., 2018; Chen et al., 2019).

The potato is the third most important food crop in the world after rice and wheat in terms of human consumption (Hameed et al., 2018), and it directly affects the yield and quality of food production. Cultivated potato is tetraploid, highly heterozygous, and vegetatively propagated. Therefore, the development of new cultivars using traditional breeding methods is a long-term effort (Zhu et al., 2020). The CRISPR/Cas system has been applied to improve the genetic breeding and agricultural characteristics of potatoes and shown great potential in accelerating breeding, increasing yield, optimizing quality, and improving stress resistance. This paper introduces the types, principles, and applications of the CRISPR/Cas system in potatoes and suggests future applications of this technology in improving the characteristics of potatoes.

## 2 CRISPR-Cas structures and mechanisms

### 2.1 CRISPR/Cas9

**FIGURE 1:**
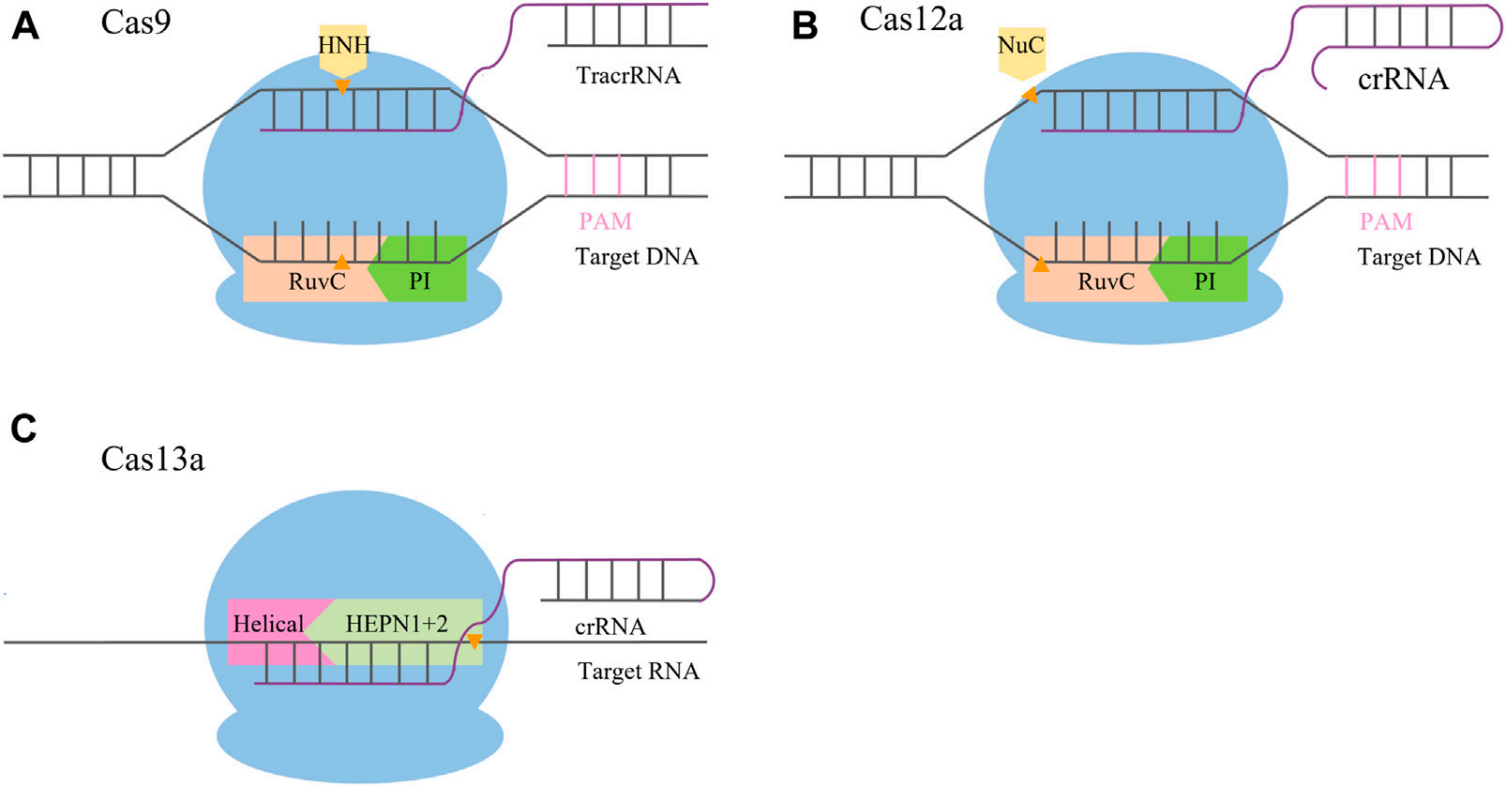
Schematic comparison of CRISPR/Cas systems. **(A)** CRISPR/Cas9 system mediates its function *via* Cas9 and two RNAs, the crRNA and tracrRNA. After hybridization, the crRNA/tracrRNA complex associates with the Cas9 nuclease and binds to its recognition site upstream of the PAM sequence. Recognition of the crRNA/tracrRNA/target complex is mediated by the REC (recognition) lobe; the PI (PAM interacting) domain is in charge of PAM recognition. The DSB is mediated by the HNH (orange) and RuvC nuclease domains (pink), with the HNH domain cleaving the target and the RuvC domain cleaving the non-target strand. **(B)** CRISPR/Cas12a system mediates its function *via* Cas12a and a single crRNA. After hybridization, the complex binds to its recognition site downstream of the PAM sequence. Recognition of the crRNA/target complex is mediated by the REC (recognition) lobe; the PI (PAM interacting, green) domain is in charge of PAM recognition. The DSB is mediated by the Nuc (orange) and RuvC domains (pink), with the Nuc domain cleaving the target strand and the RuvC domain cleaving the non-target strand. **(C)** CRISPR/Cas13 system mediates its function *via* Cas13 and a single crRNA. After hybridization, the complex binds to its recognition site within the target RNA mediated by the guide sequence of the crRNA. Recognition of the crRNA/target complex is mediated by the REC (recognition) lobe; the target RNA is cleaved by the HEPN domain (light green).

The CRISPR/Cas9 system is a complex formed by guide RNA (gRNA) and endonuclease (Cas9). gRNA is a single-stranded RNA with a specific structure (Hille et al., 2018) that can combine with the target gene and guide the Cas protein to cut the target gene. Cas9 enzymes contain an HNH domain that cleaves the DNA strand that is complementary to the guide RNA sequence (target strand) and a RuvC nuclease domain required for cleaving the non-complementary strand (non-target strand), yielding double-strand DNA breaks (DSBs) (Jinek et al., 2014) (Figure 1A). In order to protect the genome from DSB damage, cells will undergo non-homologous end joining (NHEJ) and homology-directed repair (HR). Among them, NHEJ is the main repair pathway, which is efficient for direct repair by inserting or deleting a few bases at the end. However, the insertion or deletion of bases is random and cannot be edited accurately (Mladenov and Iliakis, 2011; Wu et al., 2013; Gantz and Bier, 2015). The HR pathway is an accurate repair mechanism that can insert specific targeted modification genes and generate accurate mutations at the cutting site for accurate editing, but the repair efficiency is low (Siebert and Puchta, 2002). Jinek et al. found that CRISPR/Cas9 could recognize a 5′-NGG-3′ protospacer adjacent motif (PAM) sequence. When the Cas9 binds with PAM and the target site pairs with the gRNA, a double-strand break (DSB) is caused between positions 17 and 18 of the 20-nt gRNA sequence (Jinek et al., 2012). This laid a theoretical foundation for studying the application of the CRISPR/Cas9 system in plants. In 2013, scientists successfully edited human cells using CRISPR/Cas9 (Cong et al., 2013; Mali et al., 2013). Subsequently, the CRISPR/Cas system was rapidly applied in the field of plants, and site-directed mutations of specific gene loci were achieved in rice, wheat, maize, *Arabidopsis*, and other plants (Li et al., 2013; Nekrasov et al., 2013; Shan et al., 2013; Matres et al., 2021).

### 2.2 CRISPR/Cas12a

The CRISPR/Cas9 system has the disadvantages of a limited selection of target sites and a high off-target rate that hinder studies using the system. The CRISPR/Cas12 system greatly expands the choice of target sites for genome editing and has significant advantages in safety and accuracy. Therefore, it may become a new gene editing technology to replace the CRISPR/Cas9 system (Dong et al., 2016; Fonfara et al., 2016; Shmakov et al., 2017). The novel CRISPR DNA endonuclease (Cpf1), also known as Cas12a protein, was first reported by the Zhang Feng lab (Zetsche et al., 2015; Wu et al., 2018) in 2015, and it was used in plant gene editing in 2016 (Endo et al., 2016). Cas12a processes its own pre-crRNA into mature crRNAs, without the requirement of a tracrRNA, making it a more minimalistic system than Cas9. Cas12a possesses an RuvC domain and a nuclease (Nuc) domain. The RuvC domain of Cas12a cleaves both the non-complementary and the complementary strands of the double-stranded DNA (dsDNA), while the Nuc domain assists in the cleavage process. Therefore, DNA is cut in the same nuclease site by Cas12a, producing a staggered DSB that promotes HDR instead of NHEJ (Shmakov et al., 2015) (Figure 1B). In addition, it was proven that the off-target rate of CRISPR/Cas12a is lower than that of CRISPR/Cas9, which can ensure precise targeting of DNA (Bandyopadhyay et al., 2020).

### 2.3 CRISPR/Cas13

The CRISPR/Cas13 system is a novel gene editing technology with fascinating prospects due to its characteristic of specific RNA targeting. The CRISPR/Cas13 system is composed of two enzymatically active higher eukaryotes and prokaryotes nucleotide-binding (HEPN) *RNase* domains. One *RNase* is responsible for crRNA preprocessing, helping to form a mature interference complex, whereas the other one has two HEPN *endoRNase* domains that mediate the precise cleavage of RNA. Generally, the Cas13 protein families have been divided into four subtypes, namely, Cas13a, Cas13b, Cas13c, and Cas13d (Cox et al., 2017). Cas13a contains a Helical-1 domain and two HEPN domains. Upon the formation of guide-target RNA duplexes, Cas13a is activated by triggering the HEPN1 domain to move toward the HEPN2 domain and subsequently bind and cleave target RNA bearing a complementary sequence (Cox et al., 2017) (Figure 1C). The CRISPR/Cas13 system has been applied to confer modest interference against RNA viruses in mammalian cells and plants (Aman et al., 2018; Freije et al., 2019).

### 2.4 Base editors

**FIGURE 2:**
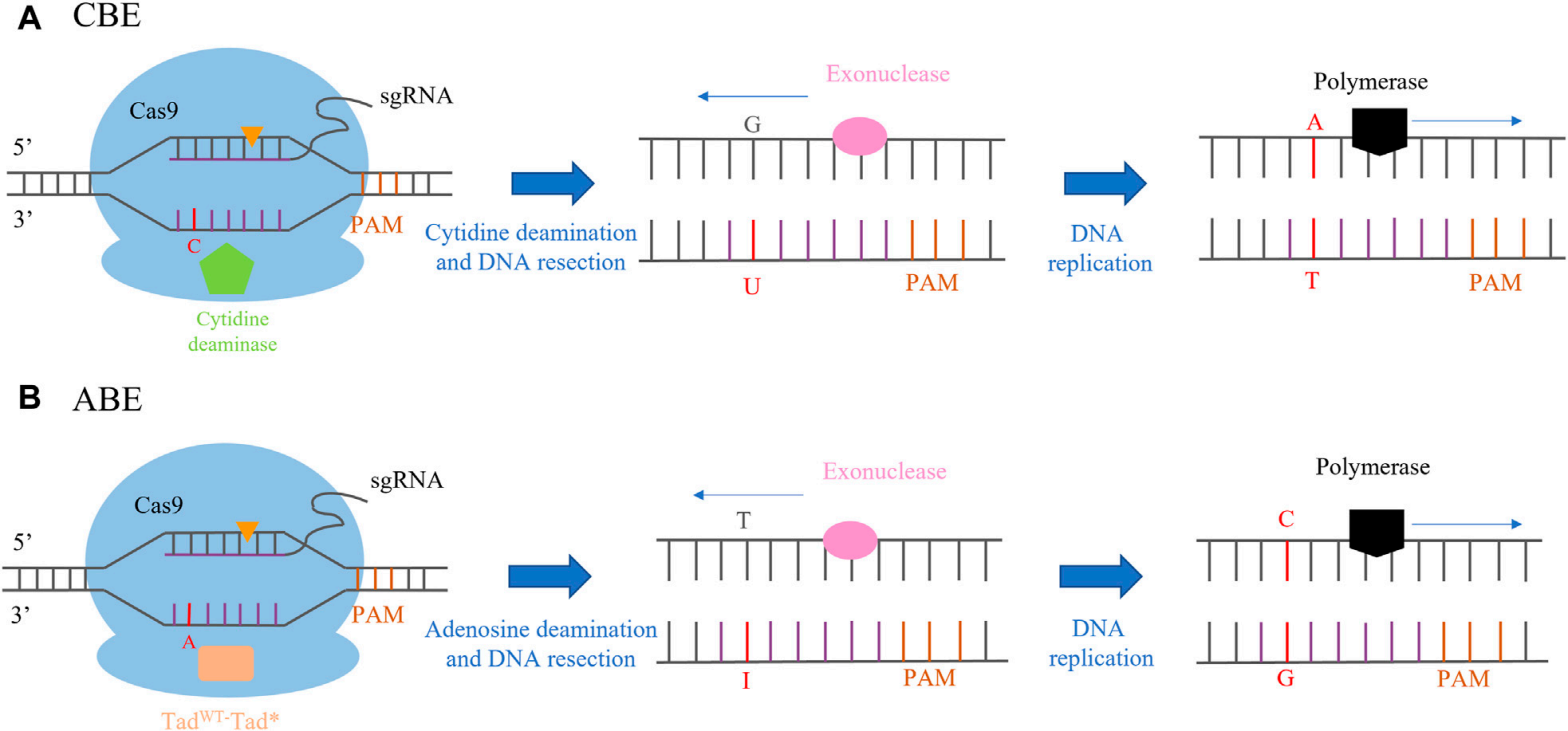
CRISPR/Cas-mediated base editing. **(A)** CBE (cytosine base editor)-mediated C-to-T base-editing strategy. Orange triangles indicate the single-stranded break within the guide RNA recognition sites. **(B)** ABE (adenine base editor)-mediated A-to-G base-editing strategy. Orange triangles indicate the single-stranded break within the guide RNA recognition sites.

The first generation of a cytosine base editor (CBE) was constructed by the fusion of rat cytosine deaminase (APOBEC) and dCas9 to form the APOBEC1-XTEN-dCas9 base editing system (Huang et al., 2021). APOBECs catalyze the deamination of cytosine bases in nucleic acids, which leads to a conversion of target cytosine (C) to uracil (U) and, consequently, a change in the single-stranded DNA/RNA sequence (Komor et al., 2016; Gaudelli et al., 2017). First-generation CBE was not effective in human cells due to cellular-mediated repair of the U-G intermediate in DNA by the base excision repair (BER) pathway. BER of U-G in DNA is initiated by uracil N-glycosylate (UNG), which recognizes the U-G mismatch and cleaves the glycosidic bond between the uracil and the deoxyribose backbone of DNA, resulting in reversion of the U-G intermediate created by the base editor back to the C-G base pair. The second generation of CBE (rAPOBEC1-XTEN-dCas9-UG) was improved by adding a uracil glycosylase inhibitor (UGI), inhibiting the activity of UDG. In the third generation of CBE, an APOBEC1-XTEN-Cas9 (D10A) base editing system was constructed by fusing the n-terminal of Rat APOBEC1 with Cas9 (D10A), which is a more efficient and convenient editing system. It has been successfully applied in crop breeding and is the most popular base editing tool at present. The fourth generation of CBE was generated by fusing an additional copy of UGI to the N-terminus of nCas9 with an optimized 27-bp linker, which greatly improved the accuracy of transformation and reduced the generation of non-target products (Gaudelli et al., 2017) (Figure 2A). The adenine base editor (ABE) is composed of the fusion of nCas9 (D10A) and adenosine deaminase. The mechanism of the ABE-mediated DNA editing operation is similar to that of CBE. The ABE-dCas9 fusion binds to a target DNA sequence in a guide RNA-programmed manner, and the deoxyadenosine deaminase domain catalyzes an adenine (A) to an inosine (I) transition. In the context of DNA replication, inosine is interpreted as guanine, and the original A-T base pair may be replaced with a G-C base pair at the target site (Gaudelli et al., 2017; Tabassum et al., 2021) (Figure 2B).

Prime editors (PEs) comprise a fusion protein consisting of a catalytically impaired SpCas9 nickase (H840A) fused to an engineered reverse transcriptase (RT) that can produce template-directed local sequence changes in the genome without the requirement for a DSB or exogenous donor DNA templates. Lin et al. adapted PEs for use in plants through codon, promoter, and editing-condition optimization (Lin et al., 2020). Anzalone et al. optimized the PEs to a “search-and-replace” genome editing technology that mediates targeted insertions, deletions, all 12 possible base-to-base conversions, and combinations thereof in human cells without requiring DSBs or donor DNA templates (Anzalone et al., 2019). Mok et al. recently developed DdCBE to enable targeted C•G-to-T•A conversions within mitochondria that have been applied for mitochondrial base editing in human embryos, mice, zebrafish, and plants. It opened a new door for base editors (Mok et al., 2022).

## 3 Applications of CRISPR/Cas in potato research

CRISPR/Cas system-mediated genome editing technology can selectively modify any genes controlling the stress resistance and nutritional quality of tuber crops to obtain desired traits. This technology plays an important role in accelerating the process of breeding, improving crop yield and quality, and enhancing stress resistance. It is a next-generation breeding technology. Potato is both an important food crop and also provides much raw material for the food processing industry (Sevestre et al., 2020). Its yield and quality are critical to global food security. The CRISPR/Cas system has been widely used in potato genetic improvement (Butler et al., 2015; Wang et al., 2015). It is an effective tool to promote the breeding of potatoes with excellent traits (Table 1).

**TABLE 1:** Applications of CRISPR/Cas system-mediated genome editing technology in potato improvement.

Trait	Editing tool	The name of target genes	Type of edit	References
Genetic breeding	CRISPR/Cas9	*Sp3* and *Sp4*	Gene knockout	Ye et al. (2018)
	CRISPR/Cas9	*S-Rnase*	Gene knockout	Taylor (2018) and Enciso-Rodriguez et al. (2019)
	CRISPR/Cas9	*StD6PK* and *StSIEL*	Gene knockout	Zhou et al. (2020) and Sun et al. (2022)
	CRISPR/Cas9	*Sli*	Gene knockout	Eggers et al. (2021)
Stress resistance	CRISPR/Cas9	*StDND1*, *StCHL1*, and *StDMR6-1*	Gene knockout	Kieu et al. (2021)
	CRISPR/Cas9	*StFLORE*	Promoter mutation	Gonzales et al. (2021)
	CRISPR/Cas9	*StCCoAOMT*	Gene knockout	Hegde et al. (2021)
	CRISPR/Cas9	*tMYB44*	Gene knockout	(Zhou et al., 2017)
	CRISPR/Cas13a	*LshCas13a*	Gene knockout	Zhan et al. (2019)
Improved quality	CRISPR/Cas9	*StGBSS*	Gene knockout	Andersson et al. (2018)
	CRISPR/Cas9	*StGBSSI*	Gene knockout	Veillet et al. (2019)
	A3A-CBE	*StGBSSI*	Base editing	Zong et al. (2018)
	CRISPR/Cas9	*StGBSSI*	Gene knockout	Wang et al. (2019)
	CRISPR/Cas9	*StGBSSI*	Gene knockout	Johansen et al. (2019)
	PmCDA1-CBE	*StGBSSI*	Base editing	Veillet et al. (2020)
	CRISPR/Cas9	*StGBSSI*	Gene knockout	Toinga-Villafuerte et al. (2022)
	CRISPR/Cas9	*SBE1* and *SBE2*	Gene knockout	Tuncel et al. (2019)
	CRISPR/Cas9	*SBE1* and *SBE2*	Gene knockout	Zhao et al. (2021)
	CRISPR/Cas9	*AtCGS* and *StMGL*	Gene knockout	Helle et al. (2018)
	CRISPR/Cas9	*St16DOX*	Gene knockout	Nakayasu et al. (2018)
	CRISPR/Cas9	*StSSR2*	Gene knockout	Zheng et al. (2021)
	CRISPR/Cas9	*StPPO2*	Gene knockout	González et al. (2019)
Improved yield	CRISPR/Cas9	*StIT1*	Gene knockout	Tang et al. (2022)

### 3.1 Overcoming the self-incompatibility of potatoes

Despite the social and economic importance of potatoes, their breeding success has remained low because self-incompatibility hindered the development of inbred lines. *S-RNase* is a key gene controlling self-incompatibility in potatoes. Ye et al. knocked out *S-RNase* using the CRISPR Cas9 system to create self-compatible (SC) diploid potatoes, which provides a new tool for diploid potato breeding (Ye et al., 2018). Meanwhile, Enciso-Rodriguez et al. also generated SC diploid lines with stable self-compatibility by targeted mutagenesis of *S-RNase* using CRISPR-Cas9 (Taylor, 2018; Enciso-Rodriguez et al., 2019). This strategy accelerates the process of diploid potato breeding and will also be useful for studying other self-incompatible crops. In addition, the haplotype-resolved genome of heterozygous diploid potatoes and tetraploid potatoes was decoded, which lays an important foundation for genome editing-assisted breeding (Zhou et al., 2020; Sun et al., 2022). The *Sli* gene was knocked out by CRISPR/Cas9 to transform SC varieties into SI varieties, and its function was studied. In addition, a 533-bp insertion fragment was found in the promoter region of the *Sli* gene, enabling it to be expressed in pollen. In future breeding work, this region can be introduced into the promoter of the *Sli* gene in SI potatoes by directional insertion to make it become an *SC* gene (Eggers et al., 2021).

### 3.2 Improving the biotic and abiotic stress resistance of potatoes

Abiotic and biotic stresses are the main factors that affect plant growth and limit agricultural productivity (Deja-Sikora et al., 2020; Sharma and Gayen, 2021). Abiotic stresses such as salinity, drought, extreme temperatures, and heavy metals (Munns and Tester, 2008; Siddiqui et al., 2017; Yang and Guo, 2018; Liu et al., 2022) are important factors affecting plant growth and development that can lead to the destruction of the original physiological characteristics of plants (Mueller and Levin, 2020). Biotic stresses of plants are caused by viral, fungal, and bacterial infections. Potatoes are usually subjected to stress from various diseases and insect pests during growth (Savary et al., 2012), such as potato early blight, late blight, ring rot, bacterial wilt, and aphids, which can lead to 30%–60% economic losses (Brown, 2011).

The CRISPR/Cas gene editing system plays an important role in accelerating the breeding process of potatoes that are highly resistant to abiotic and biotic stresses. Zhou et al., (2017) obtained the *tMYB44* mutant through the gene editing system and demonstrated that this gene negatively regulates phosphate transport in potatoes by inhibiting the expression of *StPHO1*. Tiwari et al. (2021) found that potato late blight resistance genes (*R3a*, *RGA2*, *RGA3*, *R1B-16*, *Rpi-blb2*, *Rpi*, and *Rpi-vnt1*) and susceptibility genes (S genes) are of great significance in enhancing resistance to pathogens and provided a theoretical basis for studying resistance to late blight genes. Kieu et al. (2021) found that functional knockouts of S genes, that is, creating *StDND1*, *StCHL1*, and *StDMR6-1* by the CRISPR/Cas9 system, increased resistance against late blight in potatoes. It is the first report of increasing resistance to late blight in potatoes by editing the S gene. Hegde et al. (2021) used a gene editing system to achieve directed mutation of the *StCCoAOMT* gene in the potato, which greatly improved potato resistance to late blight. Gonzales et al. (2021) mutated the *StFLORE* promoter through CRISPR/Cas9 and detected a correlation between the transcription factor *StCDF1* and the antisense transcript *StFLORE* enhancing drought resistance. The mutant could better regulate the size and number of stomata to enhance drought resistance. CRISPR/Cas13a is an RNA-targeting CRISPR effector that provides protection against RNA phages. Zhan et al. reported the repurposing of CRISPR/Cas13a to protect potato plants from a eukaryotic virus, potato virus Y (PVY), by designing sgRNAs against conserved coding regions of three different PVY strains. The levels of viral resistance correlated with the expression levels of the Cas13a/sgRNA construct in the plants. This work showed the potential of the CRISPR/Cas13a system to confer stable resistance to an important viral disease in a major crop (Ji et al., 2015; Zhan et al., 2019).

### 3.3 Improving the tuber quality of potatoes

For potatoes, a pure amylopectin starch has advantages in facilitating sustainable downstream processing with decreased use of chemicals and energy compared to native starch. CRISPR-Cas9 was developed as a potato breeding method by implementing RNP delivery in protoplasts to decrease or eliminate the presence of unintended inserts in progeny. Andersson et al. created amylopectin starch potatoes by knocking out the gene of granule-bound starch synthase (GBSS) through this method, which shows the potential of CRISPR-Cas9 RNP technology as a future potato breeding method (Andersson et al., 2018). Another strategy to develop an amylopectin potato was described soon afterward, where the use of base editing (BE) to knock out the amylose-producing *StGBSSI* gene was found successful. Amylopectin starch is most likely the most progressed trait developed with genome editing in potatoes, and non-transgenic amylopectin lines are currently grown in the field for selection and seed multiplication (Veillet et al., 2019). Many other studies report using *GBSS* as a target gene to reduce amylose content (Zong et al., 2018; Johansen et al., 2019; Tuncel et al., 2019; Wang et al., 2019; Veillet et al., 2020; Zhao et al., 2021; Toinga-Villafuerte et al., 2022).

SS6, a recently discovered starch synthase isoform, was identified as a key enzyme of the starch biosynthetic pathway (Helle et al., 2018). Sevestre et al. used a BE genome editing system to knock out the SS6 gene in potatoes. The inactivation of this enzyme may lead to modifications of starch properties, potentially resulting in industrial applications (Helle et al., 2018). Apart from its nutrients, potato also contains some anti-nutrients, such as nitrates V, toxic glycoalkaloids, and nitrates III, which can damage its quality. Steroidal glycoalkaloids (SGAs) existing in most potato tissues confer a bitter taste and show toxicity when the fresh weight is over 200 mg kg^−1^. Therefore, controlling the SGA levels in the tubers is an important focus of potato breeding. *St16DOX* encodes a steroid 16α-hydroxylase in SGA biosynthesis, which exists as the single gene in the potato genome. Therefore, this is a preferable target for genome editing to generate an SGA-free potato. Nakayasu et al. knocked out *St16DOX* through CRISPR/Cas 9, resulting in the complete abolition of the SGA accumulation in potato hairy roots (Nakayasu et al., 2018). These results provided a reference for CRISPR/Cas9 to create crops without the potential gene loci of SGAs. Potato tubers and roots are also rich in SGAs. Zheng et al. conducted targeted mutagenesis of the sterol side chain reductase 2 gene (*StSSR2*) using the CRISPR/Cas9 system. The results revealed that the SGA level in leaves and tubers was decreased by 66% and 44%, respectively. In addition, the relative transcript levels of genes involved in SGA biosynthesis pathways were also reduced (Zheng et al., 2021). Enzymatic browning catalyzed by polyphenol oxidases (PPOs) leads to the formation of dark-colored precipitates in potatoes, causing undesirable changes in organoleptic properties and the loss of nutritional quality. González et al. induced mutations in the *StPPO2* gene using a CRISPR/Cas9 system in the tetraploid cultivar Desiree. The result showed that specific editing of the *StPPO2* gene resulted in a reduction of up to 69% in tuber PPO activity and a reduction of 73% in enzymatic browning, compared to the control, which demonstrates that the CRISPR/Cas9 system can be applied to develop potato varieties with reduced enzymatic browning in tubers (González et al., 2019).

The nutritional quality of potatoes can be improved from two aspects. On one hand, the expression of the potato nutrient synthesis gene is enhanced by gene editing technology so as to improve potato nutrient content. On the other hand, adverse substances in potatoes can be reduced by knocking out the genes of potato anti-nutritional compounds and toxins (Clasen et al., 2016). Improving the nutritional value of potatoes through gene editing technology can help potatoes become a dietary staple in many countries.

### 3.4 Improving yield of potatoes

The United Nations has long listed potatoes as one of the four main foods along with corn, wheat, and rice. Potatoes have been a staple food in Western developed countries for 50–60 years. Potatoes have a great potential to increase production, nutritional value, good taste, and storage; they have a long industrial chain, and strong processing and conversion ability. The use of biotechnological tools to improve potato yields has been increasingly reported. The exogenous sucrose phosphate synthetase gene was successfully introduced into the potato, which improved the supply of photosynthates from leaves (source) to tubers (pool), thus improving the yield and quality of potato tubers (Ishimaru et al., 2008). Tuber yield was increased by inserting purple acid phosphatase 2 of *Arabidopsis* (*AtPAP2*) (Zhang et al., 2014). The successful insertion of the *Agrobacterium auxin* biosynthesis gene increased the content of indoleacetic acid in tubers and tuber formation, thus increasing yield (Kolachevskaya et al., 2015). Tuber yield can be improved by down-regulating the sucrose transporter 4 (*stsu4*) gene, the negative regulator of tubers (Chincinska et al., 2008). Tang et al. proved that the *StIT1* gene was mutated by CRISPR/Cas9, resulting in stolon branching, which laid a foundation for increased yields (Tang et al., 2022).

## 4 Future prospects

With the growth of world population, people pay more attention to food crops. Potato is one of the important food crops and has obvious advantages over other food crops in terms of per-mu yield, cost, and cultivation conditions (Zaheer and Akhtar, 2016). However, there are some major challenges facing potato production. The excellent traits of potato clones are difficult to maintain through sexual reproduction, due to the high heterozygosity in the tetraploid potato genome (Loebenstein, 2006; Nadakuduti et al., 2018). Various biotic and abiotic stresses may cause crop failure and yield loss (Xu and Gray, 2020); the toxic or anti-nutritional compounds in potatoes affect their consumption and processing (Scholthof et al., 2011; Wang et al., 2011). Classical improvement schemes comprise relatively long breeding cycles and are dictated by the genetic complexity and the sensitivity of potato to inbreeding depression. In order to meet the increasing demand for potato production, more efficient approaches for potato breeding are required. The CRISPR/Cas system, as the mainstream genome editing technique, can accelerate plant breeding by providing the means to modify genomes rapidly in a precise and predictable manner (Li M R et al., 2016; Li S C et al., 2016; Luo et al., 2016; Zhang et al., 2016; Liu et al., 2017; Lu et al., 2018; Zhang et al., 2018) and has achieved remarkable results in crop breeding. The CRISPR/Cas system has been applied to improving potato yield, quality, stress resistance, genetic breeding, and agricultural characteristics of potatoes and has shown great potential in accelerating breeding, increasing yield, optimizing quality, improving stress resistance, acquiring herbicide resistance, and reducing postharvest nutrient loss (Braatz et al., 2017; Li et al., 2017; Dong et al., 2018; Yao et al., 2018). The availability of the potato genome sequence allows scientists to precisely design its genome for crop improvement, which will facilitate the application of gene editing technology. In addition, the major genes that determine many important traits have been discovered or identified in wheat (Zong et al., 2017), maize (Svitashev et al., 2016; Chen et al., 2018; Teng et al., 2020; Jiang et al., 2021; Naik et al., 2022), rice (Zhou et al., 2019; Kuang et al., 2020; Tang et al., 2021), and soybean (Wang et al., 2020; Cai et al., 2021). The homologous genes of these genes in potato could be found and edited by the CRISPR/Cas system to construct transformants with expected traits.

The CRISPR/Cas9 gene editing system, while efficient, is not precise, leaving it vulnerable to knocking out a gene and producing many unwanted results. If the goal is to optimize the function of genes, rather than simply suppress or knock them out, the lack of predictability of the CRISPR/Cas9 system makes gene editing less feasible. Many important agronomic traits in plants are caused by single or a few base mutations. Single-site mutations in plants can be obtained based on traditional chemical mutagenesis, but this method is time-consuming, labor-intensive, random, and has a low mutation efficiency. In potato trait improvement, base editing technology can not only generate functionally acquired variants by changing individual bases but also achieve targeted evolution of specific genes by constructing sgRNA libraries to generate a library of mutations for a single gene. Ultimately, the process of potato genetic breeding is accelerated.

Although gene editing technology has great potential in agricultural production, attitudes toward the technology vary globally. To avoid falling behind the rest of the world in the gene-editing race, more than 15 countries, including China, India, Argentina, and Australia, have open rules for crop gene-editing, but these countries distinguish gene-edited crops from conventional breeding products. Many countries, such as the United States, Canada, Brazil, and Japan, do not distinguish gene editing from traditional breeding. With the development of gene editing technology, it is reasonable that more countries will remove restrictions on gene editing in the future. Different parts of the plant may have different safety risks. As a root crop, the safety risks of transgenic potato need to be further determined.

The CRISPR/Cas system has already made significant gains in potato breeding, and we expect that this is just the beginning, with many more exciting developments to follow. With the development of second-generation sequencing, gene editing technology and target analysis technology based on the high-throughput sequencing method have a solid technical foundation, and the acquisition of high-throughput big data has become more common, convenient, and affordable. These advances will greatly promote the application of CRISPR/Cas in potato genetic improvement.
